# Loofah sponge carriers: Uncovering the mechanisms of enhanced anammox performance in low-nitrogen wastewater treatment

**DOI:** 10.1016/j.isci.2026.116070

**Published:** 2026-07-27

**Authors:** Jie Luo, Jiang Fan, Hong Liu, Xintao Lv, Zhiqiang Tang, Xiaoxia Wang, Fangjiao An, Yongzhi Chen

**Affiliations:** 1 School of Environmental and Municipal Engineering, Lanzhou Jiaotong University, Lanzhou, China; 2 Technical Center of Wastewater Treatment Industry in Gansu, Lanzhou, China; 3 Ministry of Education Engineering Research Center of Water Resource Comprehensive Utilization in Cold and Arid Regions, Lanzhou, China; 4 Gansu Province Transportation Planning, Surveydesign Institute Co., Ltd, Lanzhou, China; 5 School of Environment and geography, Qingdao University, Qingdao, China; 6 School of Civil Engineering, Lanzhou University of Technology, Lanzhou, China

**Keywords:** Loofah sponge, Low nitrogen wastewater, Carrier biofilm, Enzyme functional gene, Transport proteins

## Abstract

This study investigated eco-friendly immobilization carriers for AnAOB to enhance nitrogen removal from low-nitrogen domestic wastewater. Natural loofah sponge was evaluated as a novel biofilm carrier, with polyurethane sponge and polyethylene carrier as references. Microbial morphology, community structure, and nitrogen metabolism-related functional genes were systematically analyzed. Although the loofah sponge biofilm possessed the lowest abundances of *Planctomycetota* (39.38%) and *Candidatus Brocadia* (38.35%), it achieved over 90% TNRE and a maximum TNRR of 0.067 kgN/(m^3^·d). The loofah sponge biofilm carrier also exhibited a denser, more uniform biofilm structure by SEM and higher relative abundances of key functional enzyme genes (*hdh*, *hzs*, *nirS*, and *nirK*) and ammonium transporter genes (*amt* and *FNT*) via metagenomic analysis. Long-term operation and typical cycle experiments validated its superior and stable anammox performance, providing a promising, sustainable, and easily applicable carrier strategy for practical anammox wastewater treatment systems.

## Highlights

• Loofah sponge was used as the biofilm carrier for AnAOB immobilization
• A loofah sponge carrier enabled 90% TNRE and a TNRR of 0.067 kgN/(m^3^·d)
• *Planctomycetota* and *Candidatus Brocadia* reached abundances of 39.38% and 38.35%, respectively
• Metagenomic insights into gene abundance related to nitrogen metabolism and transport proteins

## Graphical abstract

**Figure:**
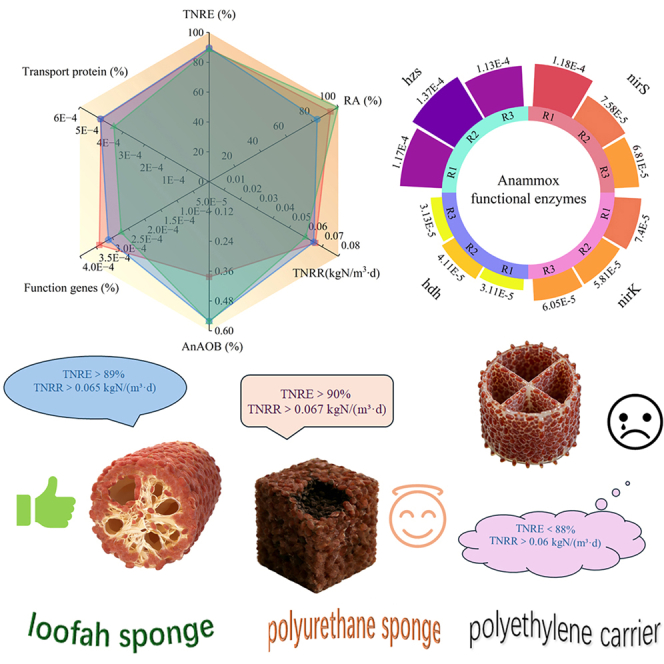

## Teaser

Biomaterials; Films

## Introduction

As the most promising biological technology for nitrogen removal from wastewater, anaerobic ammonium oxidation (anammox) can directly convert NH_4_^+^-N (electron donor) and NO_2_^−^-N (electron acceptor) into N_2_ under anaerobic conditions.^1^^,^^2^ It has the advantages of low energy consumption, no need for additional carbon sources, and low sludge and secondary pollutants production.^3^^,^^4^ The anammox technology, which has already been applied in the treatment of side-stream wastewater from wastewater treatment plants and high-concentration industrial wastewater, is also a highly valuable solution for nitrogen removal from low-nitrogen wastewater such as domestic wastewater.^5^^,^^6^

However, anammox bacteria (AnAOB) are chemoautotrophic bacteria with a slow growth rate (generation cycle approximately 11 days) and are prone to loss.^7^ Therefore, carrier immobilization is a major technique for maintaining the concentration of functional bacteria in the reactor, resisting external adverse conditions, and ensuring nitrogen removal performance.^8^^,^^9^ The immobilization carriers of microorganisms can be mainly divided into synthetic carriers and natural carriers: synthetic carriers such as polyurethane sponge and polyethylene carrier,^10^^,^^11^^,^^12^ have high mechanical strength and stable pore structure, but they also have problems such as high cost, poor biocompatibility, and the potential for secondary pollution (such as the release of microplastics)^13^^,^^14^^,^^15^; natural carriers such as straw and coconut shell fibers, although environmentally friendly, often have the defects of low porosity and easy decomposition leading to carrier loss.^16^

The loofah sponge is the dried vascular bundles of the mature luffa plant. Compared with rice straw and coconut coir fiber, loofah sponge shows distinct advantages including a unique 3D interconnected porous network, high specific surface area, stable mechanical properties, excellent mass transfer efficiency, sustained carbon release, and natural antibacterial activity, which make it a highly competitive candidate for dynamic water treatment reactors, biofilm carriers, and adsorbents; in contrast, rice straw is cost-effective but limited by poor stability and uncontrolled carbon release, thus only suitable for short-term low-load applications, while coconut coir fiber presents superior durability and mechanical strength yet insufficient pore connectivity and carbon supply capacity, making it more applicable for long-term outdoor and wear-resistant conditions. Moreover, it is widely available and low-cost and has demonstrated its potential in many fields.^17^ The loofah sponge has already been studied for use in other carriers, for example: ecological remediation,^18^ removal of antibiotics,^19^ and degradation of organic matter.^20^ However, there has been no report on the research of using the loofah sponge for the immobilization of AnAOB in the treatment of low-nitrogen domestic wastewater. The differences between loofah sponge carriers and synthetic carriers in terms of nitrogen removal performance, characteristics, microbial community structure, and metabolic mechanism still require systematic exploration.

**Figure 1:**
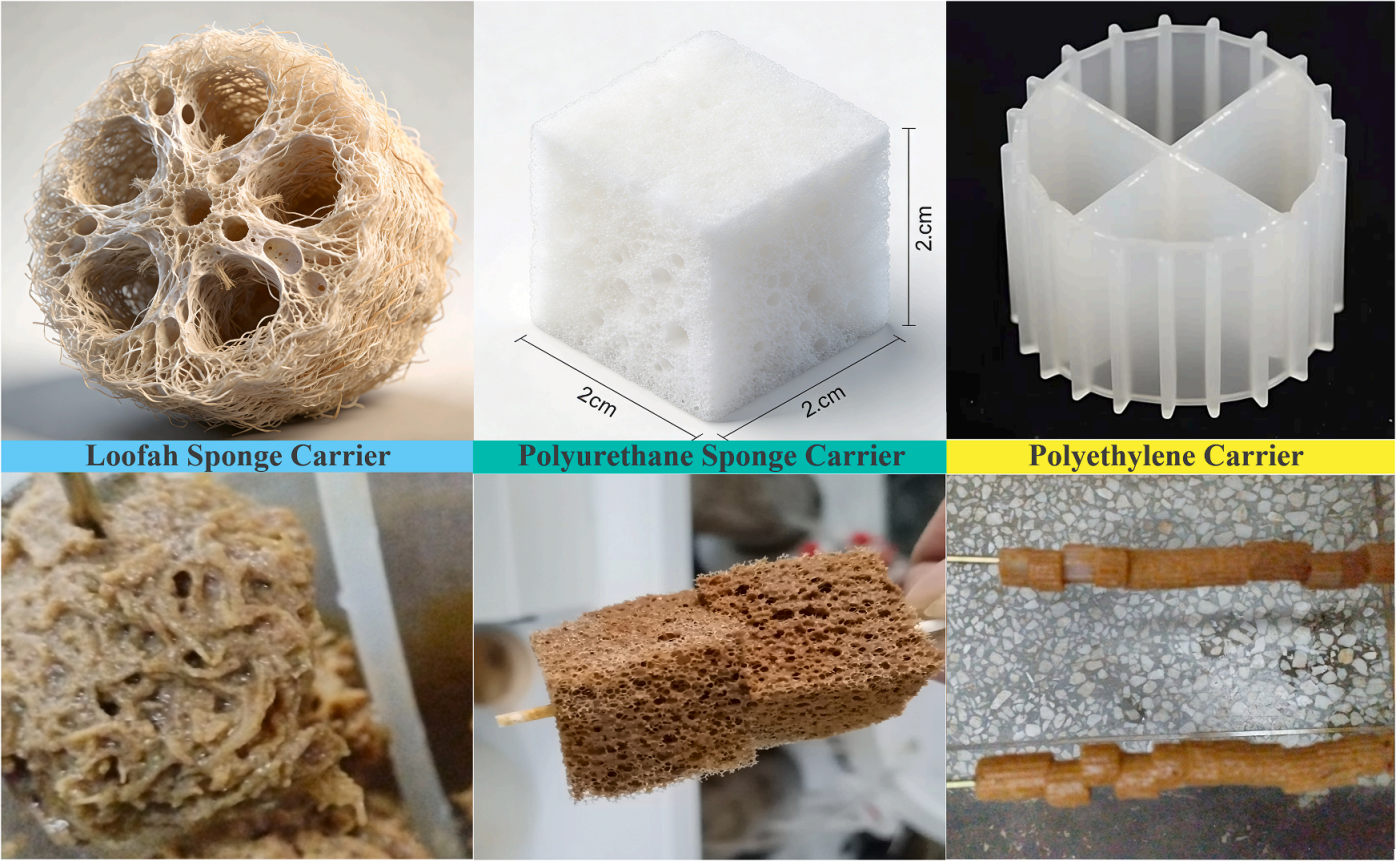
The photograph of three biofilm carriers

In this study, the synthetic low-nitrogen wastewater was used as the influent for the reactor as simulated domestic wastewater. Loofah sponge was selected as the new anammox biofilm carrier, and two commonly used synthetic carriers, polyurethane sponge^21^ and polyethylene carrier,^22^ were chosen as the control groups (Figure 1). The physicochemical properties and economic characteristics of the three biofilm carriers are compared and summarized in Table 1. The removal nitrogen efficiency of anammox after forming stable biofilms on the three immobilized carriers was investigated. The morphological, community composition, and gene expression of microorganism on the carrier surfaces were exhibited using scanning electron microscopy, high-throughput sequencing and metagenomics. The advantages of loofah sponge were analyzed from four perspectives: nitrogen removal efficiency, the increase in extracellular polymeric substances, changes in microbial abundance, and the expression of functional genes. The aim of this study was to provide new carrier options and theoretical support for the efficient and low-cost removal of nitrogen from low-nitrogen domestic wastewater.

**Table 1:** The physicochemical properties and economic characteristics of the three biofilm carriers

Performance indicator	Loofah sponge carrier	Polyurethane sponge carrier	Polyethylene carrier
Porosity	79%–93% (3D interconnected pores, 10–500 μm)	≥95% (open-cell, up to 99%, 0.2–2 mm)	35%–90% (MBBR carriers up to 90%)
Hydrophilicity	naturally hydrophilic, fast water absorption	hydrophobic unmodified; hydrophilic after modification	hydrophobic unmodified; hydrophilic after modification
Specific surface area	1.2–3.5 m^2^/g (natural)	>6,000 m^2^/m^3^ (water treatment fillers)	500–1,000 m^2^/m^3^ (MBBR); up to 8.3 m^2^/g (modified)
Surface contact angle	72.83°–76.91° (natural hydrophilic)	113° (unmodified); 0° (hydrophilic modified)	94°–110° (unmodified); 20°–82° (modified)
Surface functional groups	rich in -OH, -COOH (cellulose-hemicellulose-lignin)	contains -OH, -COOH, -NH_2_ (modifiable)	inert; modifiable to introduce -OH, -C=O, -COOH
Economic cost	very low (agricultural waste: ∼100–500 CNY/ton)	medium-high (750–1,980 CNY/m^3^)	medium (∼2,300–2,500 CNY/m^3^)

## Results

Please see Table 2.

**Table 2:** The operation parameters during each stage

Phase	Reactor	Volume	Time	NH_4_^+^-Ninf	NO_2_^−^-Ninf	HRT/operation times
		L	d	mg/L	mg/L	h
Ⅰ	continuous-flow column anaerobic reactor	5	0–80	35	46.2	8
Ⅱ	anaerobic sequencing batch reactor (ASBR)	1	81–110	35	46.2	8

### The performance and characteristics of nitrogen removal in biofilm cultivation stage

**Figure 2:**
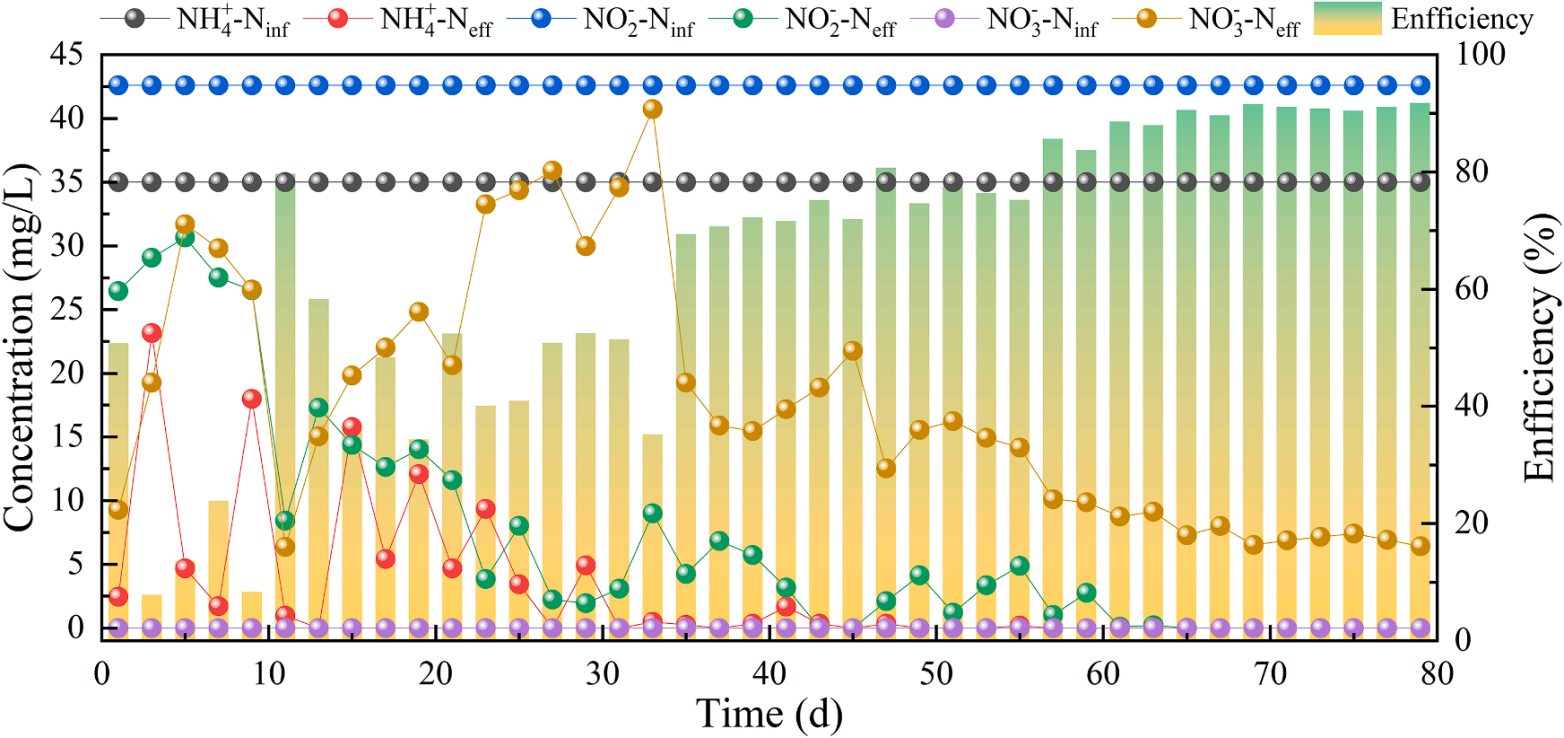
The effect of removal nitrogen in the cultured of biofilm

The biofilm cultivation stage was from 0 to 80 days. To ensure consistent conditions for the cultivation process, the three types of carriers were placed in the same continuous-flow reactor. As shown in Figure 2, the TNRE in the reactor was relatively low from 1 to 10 days, increased from about 20% to about 40% from 10 to 35 days, and then sharply rose to 70% on the 35th day, entering a stable and steady increase period until it reached 90% on the 68th day and remained stable at over 90% until the 80th day. Before day 35, the relatively low and fluctuating TNRE was primarily attributed to significant fluctuations in NH_4_^+^-N concentration, a steady decrease in NO_2_^−^-N, and consistently high but variable NO_3_^−^-N levels in the effluent.^23^

### The nitrogen removal performance of three types of carrier biofilms in their respective reactors

**Figure 3:**
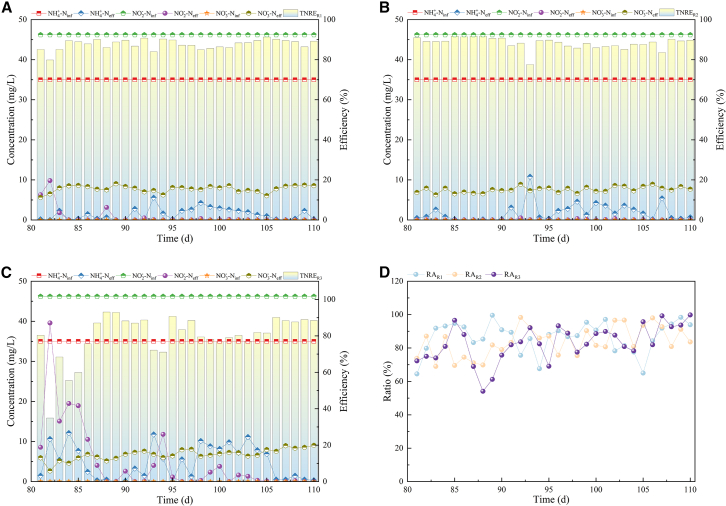
The removal nitrogen effect of biofilm in the different carriers (A) Reactor R1. (B) Reactor R2. (C) Reactor R3. (D) RA of three reactors.

As shown in Figures 3A–3C, during days 81–110, the effluent NO_3_^−^-N concentrations of the three reactors showed no significant fluctuations, with average values of 7.78, 7.58, and 6.77 mg/L, respectively. The average effluent NH_4_^+^-N concentrations were 1.43, 1.86, and 4.77 mg/L, respectively. Both R1 and R2 exhibited slight fluctuations between days 90 and 105, with more pronounced variability observed in R2. In contrast, the effluent ammonia nitrogen concentration in R3 fluctuated during almost the entire monitoring period and only stabilized after day 106.

The average effluent nitrite concentrations were 0.75, 0.06, and 4.87 mg/L, respectively; R2 performed better than R1, and R3 was the worst. However, the effluent NO_2_^−^-N concentration in R1 was higher than that in R2 during the first four days, and its performance was basically consistent with R2 in the later stage, with NO_2_^−^-N being almost completely removed. In comparison, the effluent NO_2_^−^-N concentration in R3 fluctuated continuously during the first 15 days, especially in the first 7 days, when the difference between effluent and influent concentrations was insignificant. During this period, the NO_2_^−^-N removal efficiency was only around 50% and did not stabilize until day 104. The effluent NO_2_^−^-N concentration in R3 tended to be stable after day 106.

The TNRE values in R1 and R2 were similar, whereas R3 performed poorly from days 80–87 and exhibited large fluctuations from days 87–105. After day 105, it also stabilized similarly to R1 and R2, achieving a TNRE of over 90%.

### The nitrogen removal efficiency of three biofilms in typical cycle

On day 110 of the experiment, a typical cycle test was conducted for the three reactors. Water samples were collected and the concentrations of NH_4_^+^-N, NO_2_^−^-N, and NO_3_^−^-N were measured at 0.5, 1, 2, 3, 4, 5, 6, and 8 h, respectively.

**Figure 4:**
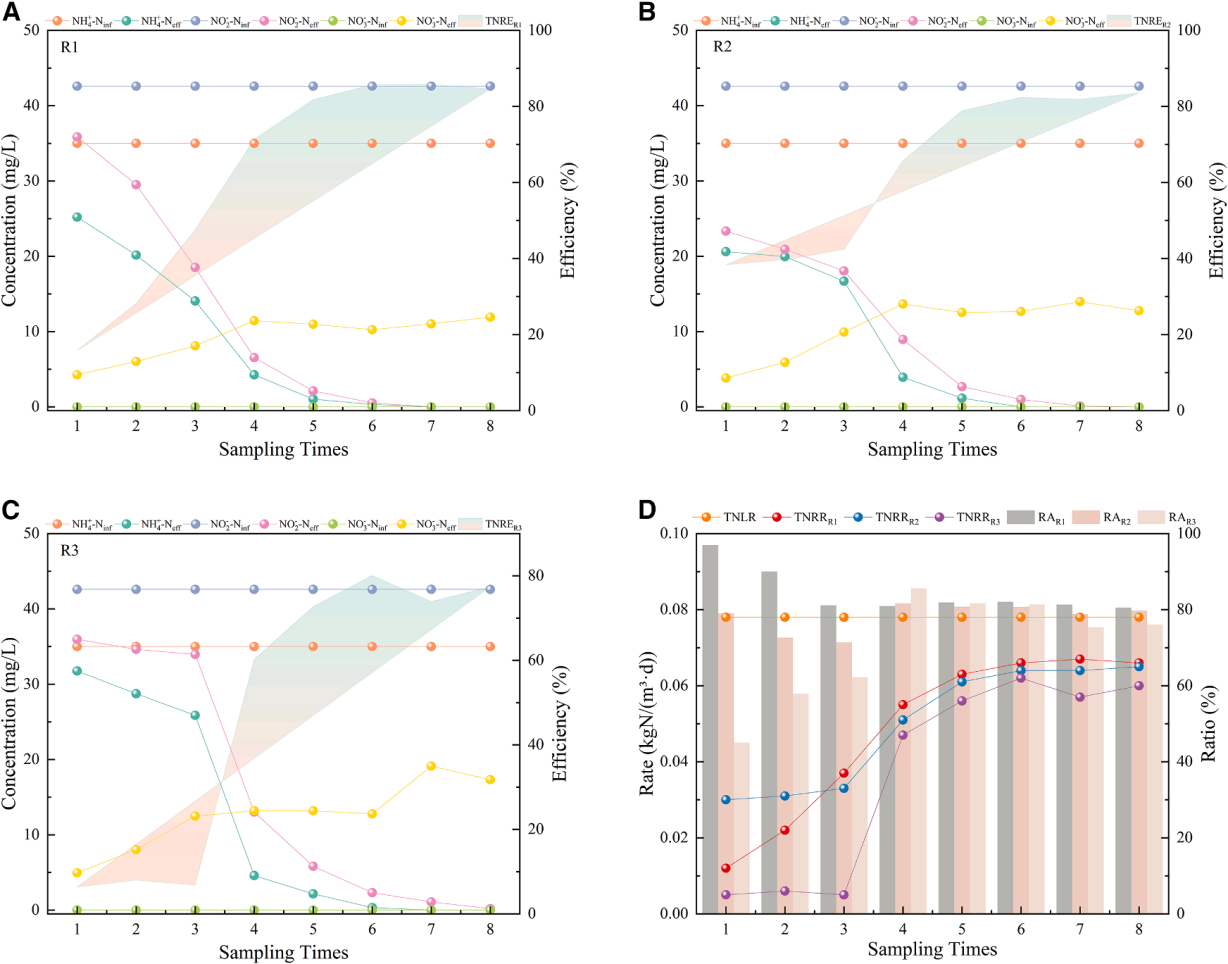
The removal nitrogen effect of three biofilms in typical cycle (A) The biofilm of loofah sponge carrier. (B) The biofilm of polyurethane sponge carrier. (C) The biofilm of polyethylene ring carrier. (D) The TNLR, TNRR, and RA in three reactors.

As shown in Figure 4, among the three reactors, the concentration of NH_4_^+^-N decreased significantly in the initial reaction stage, falling below 5 mg/L within 5 h, and NH_4_^+^-N was almost completely removed at approximately 16 h. However, the removal of NO_2_^−^-N was most efficient in R1, followed by R2, and least efficient in R3. At 4 h, the residual amount of NO_2_^−^-N in R1 was 27.1% lower than in R2 and 49.7% lower than in R3. As the reaction proceeded, NO_2_^−^-N in R1 and R2 was almost completely removed, whereas a small amount of residual NO_2_^−^-N remained in R3 after 24 h. The increasing trend of NO3^−^-N concentration was similar in R1 and R2, but in R3, it increased significantly after 12 h, exceeding the NO3^−^-N yield from anammox as predicted by classical theory.

At initial stage of reaction, the TNRE was the highest in R2 in three reactors, but R1 first surpassed R2 at 2 h and subsequently maintained and extended this advantage. At 16 h, the TNRE of R1 reached a peak of 85.79%, which was 3.9% higher than that of R2 and 15.9% higher than that of R3. Ultimately, at the 24th hour, the TNRE of R1 was 84.63%, which demonstrated significantly better stability than R2 (83.55%) and R3 (77.44%).

As shown in Figure 4D, the RA in reactor R1 remained the highest throughout the entire process. It peaked at 97.02% at 0.5 h, which was 22.7% higher than that in R2 and 2.15 times that in R3. Over the entire 24 h period, the RA in R1 still maintained the highest value at 85.03% providing a solid foundation for the anammox reaction.

According to the experimental design of this study, the system TNLR value was determined to be 0.078 kgN/(m^3^·d). Although the initial TNRR of R1 was lower than that of R2, it surpassed R2 at 2 h and steadily increased. At 16 h, the TNRR of R1 reached a peak of 0.067 kgN/(m^3^·d), which was 4.7% higher than that of R2 and 17.5% higher than that of R3. At this time, the nitrogen removal efficiency of the R1 reactor reached 85.90%. Furthermore, no significant attenuation was observed from 16 to 24 h.

### The characteristics of microorganisms under scanning electron microscopy

Observations of the microscopic morphology of biofilms via scanning electron microscopy (SEM) at magnifications of 5,000×, 10,000×, and 30,000× directly reflect the effects of carrier properties on biofilm formation, microbial adhesion, and community structure. Integrating the SEM imaging data, differences in biofilms across the three carriers were compared.

**Figure 5:**
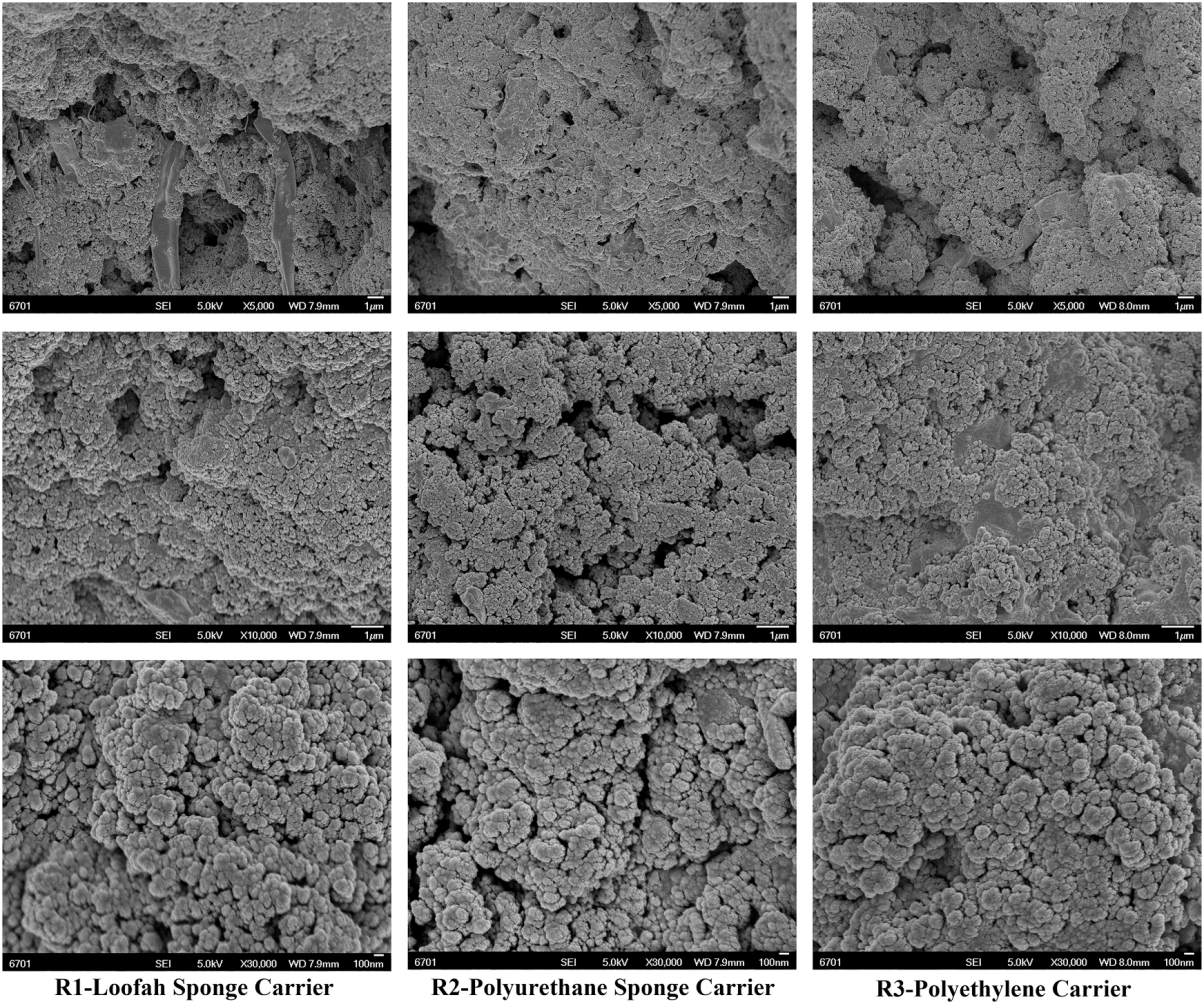
Representative SEM images of biofilm on three carriers at different magnifications Scale bars, 1 μm (×5,000 and ×10,000 magnifications); 100 nm (×30,000 magnification). (R1) Loofah sponge carrier; (R2) polyurethane sponge carrier; (R3) polyethylene carrier.

According to Figure 5, SEM micrographs at 5,000× clearly reveal the overall coverage of biofilms on carrier surfaces and the filling characteristics of carrier pores. Specifically: the surface and interstices of the fibrous interwoven network of the R1 (loofah sponge) carrier are densely populated with microorganisms, with the biofilm tightly adhering to the carrier framework; a dense microbial film is attached to the surface of the R2 (polyurethane sponge) carrier; the R3 (polyethylene ring) carrier exhibits localized thickening and accumulation of biofilm on its surface, with relatively large and deep pores formed between microorganisms, indicating uneven biofilm thickness.

At 10,000× magnification, the typical oval morphology of AnAOB can be initially identified. Significant differences in microbial distribution were observed among the three carriers: microorganisms on the R1 carrier surface are uniformly distributed with regularly arranged bacterial cells; the R2 carrier retains a thick biofilm accumulation state, with pores of a certain depth between cells, and microbial adhesion is also detectable within these pores; the R3 carrier shows heterogeneous microbial distribution, with the polyethylene surface exposed beneath the biofilm layer in multiple regions, indicating that the biofilm layer is thin and discontinuous.

At 30,000× magnification, microorganisms on all three carriers form dense oval biofilm layers, which are generally consistent with the typical characteristics of AnAOB, but distinct differences in microstructure were noted: the R1 carrier exhibits relatively compact bacterial floc structures with numerous internal pores, suggesting a layered distribution of different bacterial morphotypes; on the R2 carrier surface, various bacterial morphologies (rod-shaped and spherical) are tightly embedded in a rich extracellular polymeric substance (EPS) matrix, with strong intercellular connections; the R3 carrier displays microbial morphologies similar to those on R1, but relatively deep pores are clearly observable, indicating localized thickening of the biofilm.^24^

### Characteristics of stratified EPS in biofilm

**Figure 6:**
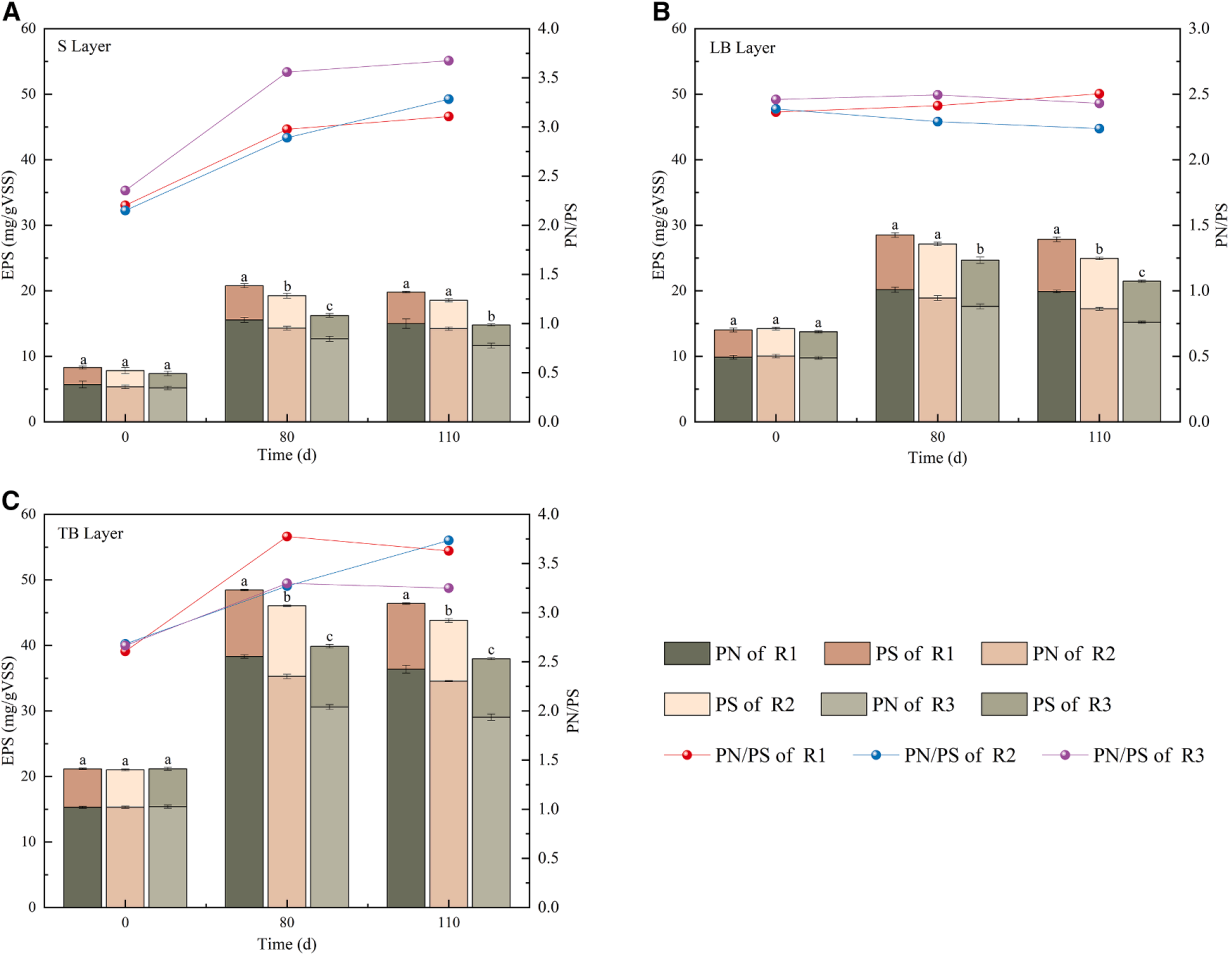
The concentrations of EPS and PN/PS in different layers of anammox biofilm at different operational stages (A) The S layer of EPS. (B) The LB layer of EPS. (C) The TB layer of EPS. Data are presented as mean ± standard error of the mean (SEM, *n* = 3). Different lowercase letters above the bars indicate significant differences among reactors (R1, R2, and R3) within the same layer and time point (*p* < 0.05, one-way ANOVA followed by Tukey’s HSD test). The lines represent the PN/PS ratio for each reactor, with red, blue, and purple dots corresponding to R1, R2, and R3, respectively.

EPSs extracted from biofilm in the three reactors were used to further analyze the role of carriers in the aggregation of AnAOB.^25^ As shown in Figure 6, the total EPS concentration in all layers increased significantly from day 0 to day 80, followed by a slight decrease or stabilization at day 110. In the S-layer (a), the total EPS concentration increased from approximately 7 mg/gVSS at day 0–20 mg/gVSS at day 80, with R1 exhibiting the highest concentration (20.77 mg/gVSS) and R3 the lowest (16.23 mg/gVSS), which was consistent with the abundance of functional gene. A similar trend was observed in the LB-layer (b) and TB-layer (c), where the total EPS concentration peaked at day 80, reaching up to 48.45 mg/gVSS in the TB-layer for R1.

All biofilms demonstrated consistent characteristics during the cultivation process: total EPS increased with biofilm maturation, driven primarily by the TB-EPS layer; the rise in protein (PN) substantially exceeded that of polysaccharide (PS), leading to an elevated PN/PS ratio. These trends align with previous similar studies.^26^

During the day 0–80, total EPS increased by 38.49–54.26 mg/gVSS in this process.

In the TB-EPS of all biofilms, the PN/PS ratio increased from 2.6 to 2.71 in the initial stage to 3.25–3.77 at maturity, and remained at 3.31–3.74 in the final stable stage, showing little change from the mature phase.

Statistical analysis revealed that, while no significant differences in total EPS concentration were observed among all reactors within each layer on day 0 (*p* > 0.05), significant differences were detected among nearly all reactors within each layer on days 80 and 110 (*p* < 0.05). Specifically, on day 80 in the LB layer and on day 110 in the S layer, no significant difference was found between reactors R1 and R2, whereas significant differences were observed between R1 and R3.

### Microbial community diversity analysis

**Figure 7:**
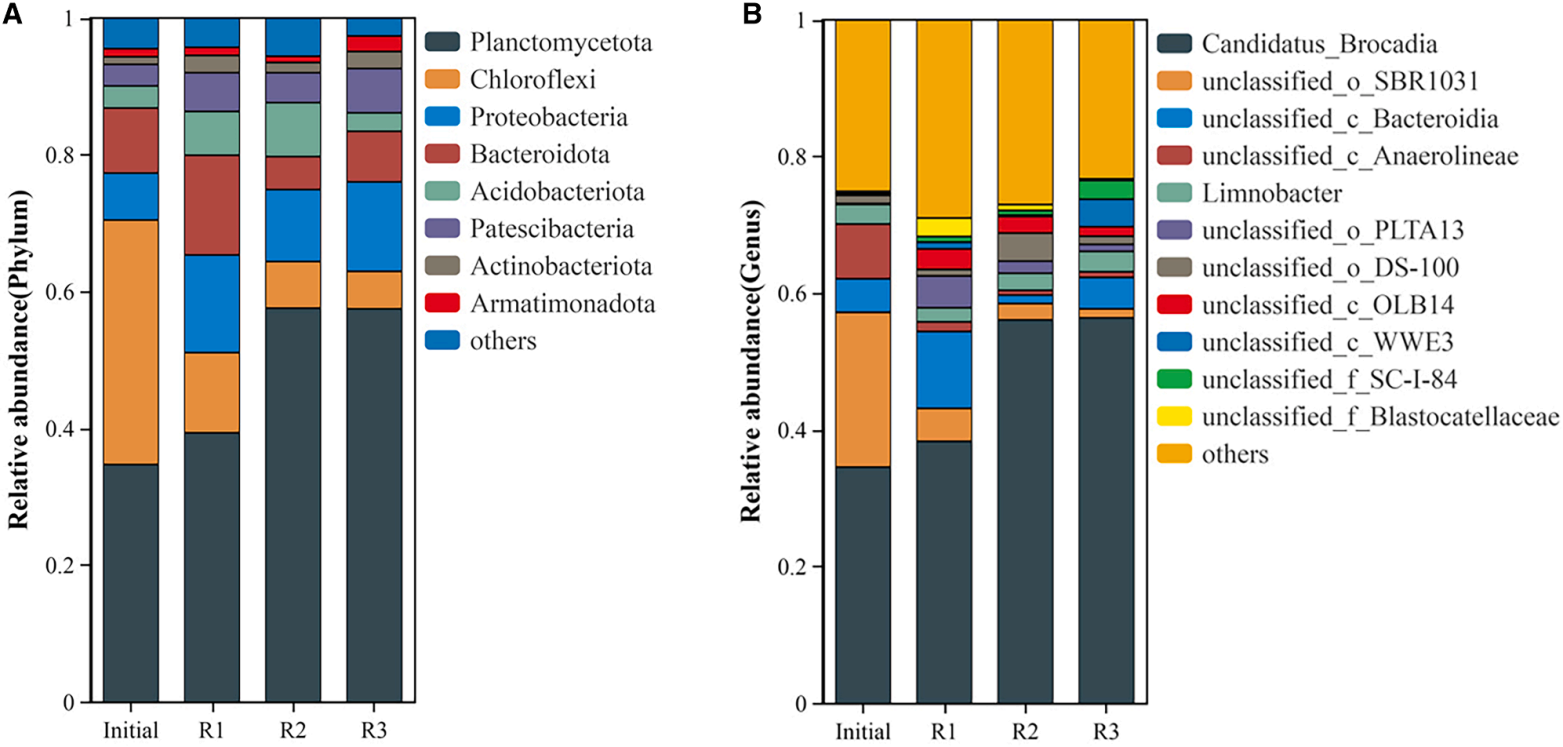
The microbial community abundance of phylum and genus levels (A) Phylum level. (B) Genus level.

As shown in Figure 7, the bar chart presents the relative abundance of major functional microbial in the phylum and genus levels in anammox suspended sludge prior to carrier biofilm cultivation and in the three reactors (R1, R2, and R3) postcultivation, derived from high-throughput sequencing of microbial 16S rRNA. Among them, Figure 7A shows the relative abundance at the phylum level. The main functional microorganisms and their respective abundances in the initial flocculent sludge and in the three reactors after biofilm cultivation are as follows: *Planctomycetota* (34.71%, 39.38%, 57.59%, and 57.41%), Chloroflexi (35.75%, 11.73%, 6.83%, and 5.49%), Proteobacteria (6.85%, 14.27%, 10.51%, and 13.07%), *Bacteroidota* (9.51%, 14.57%, 4.81%, and 7.39%), *Acidobacteriota* (3.24%, 6.40%, 7.90%, and 2.70%), *Patescibacteria* (3.14%, 5.66%, 4.36%, and 6.47%), *Actinobacteriota* (1.11%, 2.52%, 1.49%, and 2.49%), and Armatimonadota (1.19%, 1.19%, 0.92%, and 2.27%). Within *Planctomycetota*, the dominant AnAOB genus is *Candidatus Brocadia*, with abundances of 34.59%, 38.35%, 56.10%, and 56.40%, respectively.^27^ For obligate anammox functional bacteria, the abundance in R1 was the lowest, increasing only from 34.59% in the initial flocculent sludge to 38.35%.

### Abundance of nitrogen metabolism and transport proteins functional genes

Metagenomics was utilized to further elucidate the changes in enzyme functional genes related to the performance of anammox during the entire three kinds of biofilms, processes where nitrogen metabolism are central microbial metabolic functions.^28^

#### Abundance of nitrogen metabolism functional genes

**Figure 8:**
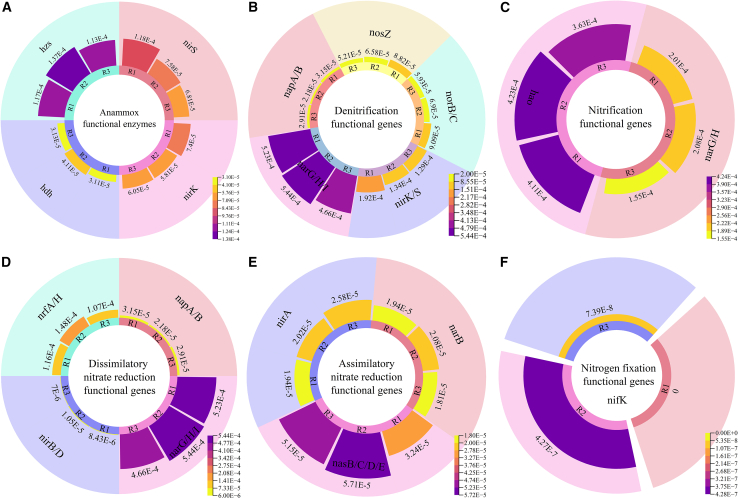
The genes abundance of functional enzymes in the nitrogen cycle pathway (A) The abundance of anammox functional enzymes. (B) The abundance of denitrification functional enzymes. (C) The abundance of nitrification functional enzymes. (D) The abundance of dissimilatory nitrate reduction functional enzymes. (E) The abundance of assimilatory nitrate reduction functional enzymes. (F) The abundance of nitrogen fixation functional enzymes.

Metagenomic KEGG sequencing results revealed six major metabolic pathways in the nitrogen cycle, namely the anammox, denitrification, nitrification, dissimilatory nitrate reduction, assimilatory nitrate reduction, and nitrogen fixation pathways.^29^ Among these, the anammox pathway is the core focus of this study. The key enzymes involved in this pathway primarily include hydrazine dehydrogenase (*hdh*), hydrazine synthase (*hzs*), and nitrite reductase (*nirS* and *nirK*).^30^^,^^31^ As shown in Figure 8A, the abundances of *nirK* and *nirS* in reactor R1 were the highest, at 7.4 × 10^−5^ and 1.18 × 10^−4^, respectively; while the abundance of *hzs* in R1 was 1.17 × 10^−4^, thus ranking second among the three reactors—with R2 exhibiting the highest abundance and R3 the lowest. In contrast, the abundance of *hdh* in R1 was 3.11 × 10^−5^, the lowest among the three reactors; R2 had the highest *hdh* abundance, followed by R3.

As shown in Figure 8B, among the five key enzyme types involved in the denitrification pathway, reactor R1 exhibited the highest abundances of nitrous oxide reductase (*nosZ*), nitric oxide reductase (*norB/C*), nitrite reductase (*nirK/S*), and nitrate reductase (*napA/B*), at 8.82 × 10^−5^, 9.09 × 10^−5^, 1.92 × 10^−4^, and 3.15 × 10^−5^, respectively. For the fifth enzyme type (*narG/H/I*), R1’s abundance was 5.23 × 10^−4^, ranking second.

As shown in Figure 8C, for the two key enzymes (*hao* and *narG/H*) involved in the nitrification pathway, reactor R1 exhibited abundances of 4.11 × 10^−4^ and 2.01 × 10^−4^, respectively, ranking second. Reactor R2 had the highest abundances of these enzymes, at 4.23 × 10^−4^ and 2.08 × 10^−4^, respectively, while reactor R3 had the lowest, at 3.63 × 10^−4^ and 1.55 × 10^−4^, respectively.

As shown in Figure 8D, the key functional enzymes involved in the dissimilatory nitrate reduction pathway include four types: *nrfA/H*, *napA/B*, *nirB/D*, and *narG/H/I*. Among these, the abundances of *nrfA/H* in R1, R2, and R3 were 1.16 × 10^−4^, 1.48 × 10^−4^, and 1.07 × 10^−4^, respectively; *napA/B* abundances were 3.15 × 10^−5^, 2.18 × 10^−5^, and 2.91 × 10^−5^, respectively; *nirB/D* abundances were 8.43 × 10^−6^, 1.05 × 10^−5^, and 7.00 × 10^−6^, respectively; and *narG/H/I* abundances were 5.23 × 10^−4^, 5.44 × 10^−4^, and 4.66 × 10^−4^, respectively. In reactor R1, *napA/B* abundance ranked first among enzymes associated with this pathway, while the abundances of the other three enzymes were relatively low.

As shown in Figure 8E, the key functional enzymes involved in the assimilatory nitrate reduction pathway include three types: *nirA*, *narB*, and *nasB/C/D/E*. The abundances of *nirA* in R1, R2, and R3 were 1.94 × 10^−5^, 2.02 × 10^−5^, and 2.58 × 10^−5^, respectively; *narB* abundances were 1.94 × 10^−5^, 2.08 × 10^−5^, and 1.81 × 10^−5^, respectively; and *nasB/C/D/E* abundances were 3.24 × 10^−5^, 5.71 × 10^−5^, and 5.15 × 10^−5^, respectively. In reactor R1, among these three functional enzyme types, only *narB* abundance ranked second; *nirA* and *nasB/C/D/E* abundances were the lowest among the three reactors.

As shown in Figure 8F, the only key functional enzyme involved in the nitrogen fixation pathway in this experiment was *nifK*, which was present only in trace amounts in R2 and R3, at abundances of 4.27 × 10^−7^ and 7.39 × 10^−8^, respectively. *nifK* abundance in reactor R1 was 0.

#### Abundance of transport proteins functional genes

In this study, to investigate their impact on the anammox reaction, we selected seven transport proteins associated with anammox and analyzed their gene expression levels across three distinct carrier biofilms. This selection comprised two key transport proteins directly involved in the anammox reaction and five auxiliary proteins closely linked to the process. The key transport proteins included ammonium transporter (*amt*),^32^^,^^33^ nitrite transporter (*FNT*),^34^ and others auxiliary transport proteins. Comparative analysis of metagenomic data against the Transporter Classification Database (*TCDB*) revealed abundance differences of these transporters gene during three carrier biofilms.

**Figure 9:**
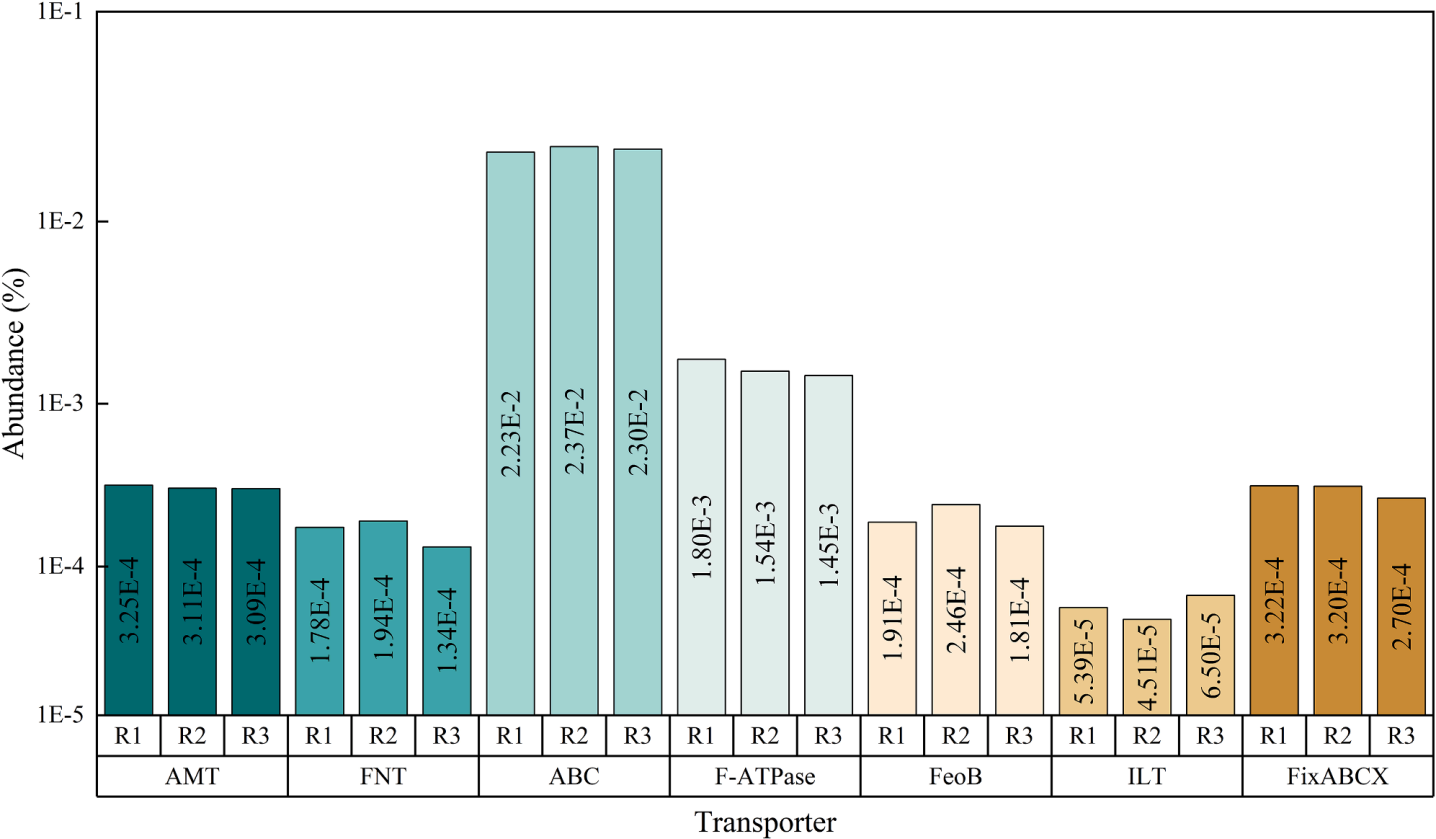
The genes abundance of transport proteins in the nitrogen cycle pathway

As shown in Figure 9, the abundance of the *amt* gene was highest in R1 (3.25E−4), followed by R2 (3.11E−4), and lowest in R3 (3.09E−4). *FNT* serves as the key channel for AnAOB to acquire NO_2_^−^, its abundance in the biofilm was the highest in R2 (1.94E−4), with R1 ranking second (1.78E−4). However, the abundances of both *amt* and *FNT* in R3 were the lowest among the three reactors.

In reactor R1, the abundances of both *FeoB* and *ILT* ranked second, being only 22.39% and 17.08% lower than their respective maximum values. The highest abundances of *FeoB* and *ILT* were detected in R2 (2.46E−4) and R3 (6.50E−5), respectively.

Among the three reactors, the abundance of *ABC* transporters in R1 was the lowest (2.23E−2); however, this value was only 5.91% lower than the highest abundance. Within the remaining auxiliary transport proteins, the *F-ATPase* (1.80E−3) and *FixABCX* (3.22E−4) proteins in reactor R1 maintained a clear functional advantage in transporting hydrogen ions and nitric oxide.^35^^,^^36^

#### Co-expression analysis of core functional genes in the anammox process

**Figure 10:**
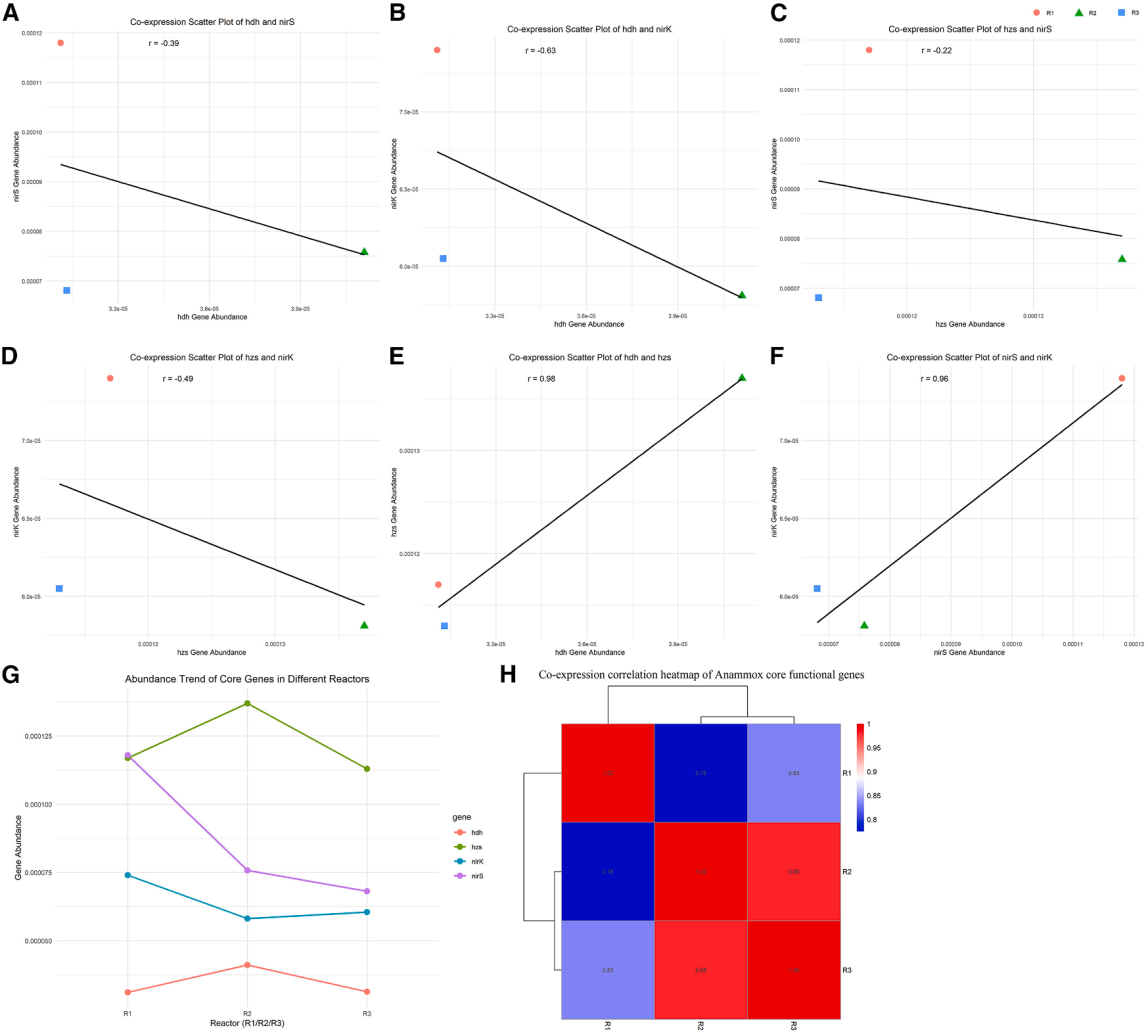
Co-expression analysis of core functional genes in three anammox reactors (R1, R2, and R3) (A–F) Pairwise co-expression scatterplots of key anammox genes (*hdh* vs. *nirS*, *hdh* vs. *nirK*, *hzs* vs. *nirS*, *hzs* vs. *nirK*, *hdh* vs. *hzs*, and *nirS* vs. *nirK*). (G) Abundance trends of core functional genes (*hdh*, *hzs*, *nirK*, and *nirS*) across reactors. (H) Co-expression correlation heatmap of overall functional gene profiles among reactors.

The pairwise co-expression relationships among four core anammox functional genes (*hdh*, *hzs*, *nirK*, and *nirS*) were evaluated using Pearson correlation analysis (Figures 10A–10F).

Negative correlations were observed between *hdh* and *nirS* (r = −0.39), *hdh* and *nirK* (r = −0.63), *hzs* and *nirK* (r = −0.49), and *hzs* and *nirS* (r = −0.22). In contrast, strong positive correlations were detected between *hdh* and *hzs* (r = 0.98) and between *nirS* and *nirK* (r = 0.96). Notably, data points from reactor R1 (red dots) consistently clustered at higher gene abundance levels for *hdh*, *hzs*, and *nirK*, while R3 (green triangles) showed lower abundance, and R2 (blue squares) occupied an intermediate position.

The total abundance of core functional genes varied significantly across reactors (Figure 10G). R1 exhibited the highest overall gene abundance, with particularly elevated levels of *hdh* and *hzs*, which are central to hydrazine synthesis and oxidation—the rate-limiting steps of anammox. R2 showed moderate abundance, while R3 displayed the lowest levels of all target genes.

As shown in Figure 10H, the co-expression correlation heatmap provides a clear view of the inter-reactor correlations in the overall functional gene expression profiles. R1 showed a strong positive correlation with R3 (r = 0.83), but a moderate positive correlation with R2 (r = 0.78). R2 exhibited very strong positive correlations with R3 (r = 0.98).

#### Correlation analysis between core functional gene expression and nitrogen removal performance

The relationships between the mean abundance of core anammox genes and key nitrogen removal performance indicators were assessed using Pearson correlation analysis.

**Figure 11:**
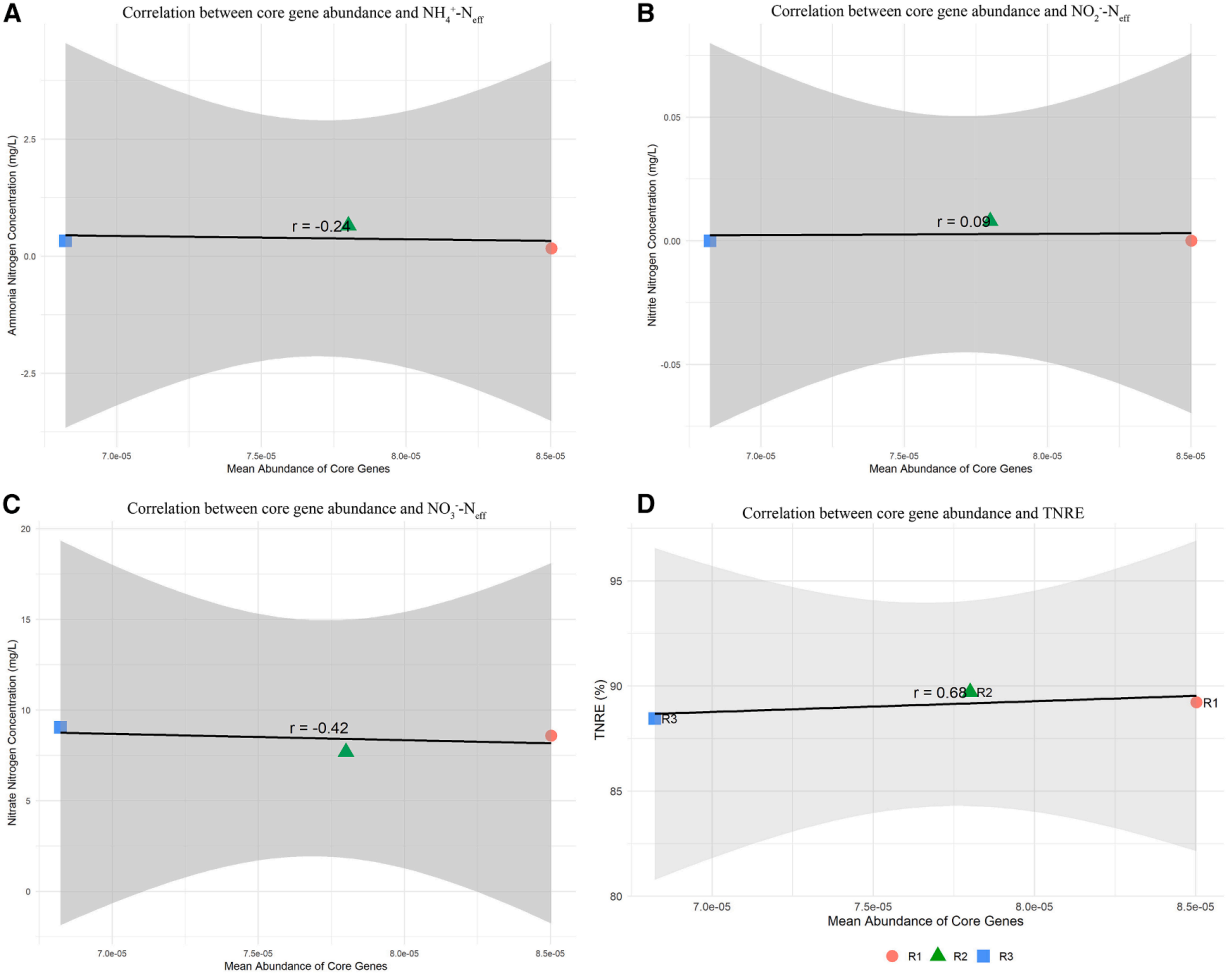
Correlation analysis between mean abundance of core anammox functional genes and nitrogen removal performance in reactors R1, R2, and R3 (A) Correlation between core gene abundance and NH4^+^-N_e_ff concentration, (B) correlation between core gene abundance and NO2^−^-N_e_ff concentration, (C) correlation between core gene abundance and NO3^−^-N_e_ff concentration, (D) correlation between core gene abundance and TNRE. Pearson correlation coefficients (R) are shown in each panel. The gray-shaded area represents the 95% confidence interval of the linear regression. Data are presented as mean values (*n* = 3).

As shown in Figures 11A–11D, a weak negative correlation (r = −0.24) was observed between core gene abundance and NH_4_^+^-N_eff_. R1 (red dot) and R3 (blue square) exhibited the highest and lowest core gene abundance, respectively, with R1 showing the lowest NH_4_^+^-N_eff_ concentration. This trend suggests that higher functional gene expression may contribute to more efficient ammonia removal.

A negligible positive correlation (r = 0.09) was detected. Notably, R1 maintained the lowest NO_2_^−^-N_eff_ concentration despite its high gene abundance, indicating effective nitrite utilization within the anammox pathway.

A moderate negative correlation (r = −0.42) was found between core gene abundance and NO_3_^−^-N_eff_. R1, with the highest gene abundance, showed the lowest NO_3_^−^-N_eff_ level, implying that enhanced functional gene expression may reduce nitrate production, a by-product of anammox metabolism.

A strong positive correlation (r = 0.68) was observed between core gene abundance and TNRE. R1 exhibited the highest core gene abundance and the highest TNRE (≈88%), while R3 showed the lowest values for both metrics. This strong correlation directly links the transcriptional activity of key anammox genes to the overall nitrogen removal capacity of the system.

## Discussion

These results of nitrogen removal in biofilm cultivation stage indicate that the microorganisms have been stably fixed on the carriers and achieved the best removal nitrogen effect. In initial stage, poor nitrogen removal effect was likely caused by the incomplete attachment and acclimation of microorganisms to the carriers under hydraulic shear stress, leading to an unstable nitrogen removal performance of the immature biofilm.^37^ Although the reactor bulk environment was predominantly anaerobic, a dissolved oxygen gradient existed within the biofilm.^38^ Residual oxygen present in the carrier gaps created localized anoxic or micro-aerobic zones, which supported limited nitrification. This explains the observed higher nitrate proportion compared to the classical anammox reaction.^8^ Ammonia-oxidizing bacteria (AOB) and nitrite-oxidizing bacteria (NOB) could survive at the carrier surface and inside or at local oxygen exposure points caused by the stirrer.^39^ NOB would oxidize part of NO_2_^−^-N to NO_3_^−^-N, which directly led to a higher NO_3_^−^-N concentration in the effluent than the theoretical value of the classical anammox reaction. Studies have shown that it is extremely challenging to completely inhibit NOB in a biofilm system because the anoxic zones within the biofilm may provide a refuge for NOB.

The organic matter produced by microbial metabolism in the water would promote the proliferation of heterotrophic denitrifying bacteria. The activities of these bacterial communities would compete with anammox bacteria for substrates (nitrite) and even ammonia nitrogen, which intensified the fluctuations in ammonia nitrogen and might temporarily inhibit the anammox process. However, some denitrifying bacteria would perform partial denitrification (PD), incompletely reducing nitrate (NO_3_^−^-N) to nitrite (NO_2_^−^-N). This provided a crucial substrate for the anammox reaction and was one of the key reasons why nitrite could be continuously consumed while nitrate fluctuated in the system.

In the initial stage of cultivation, the inoculated flocculent anammox bacteria needed time to attach, colonize, and proliferate on the carriers. At this stage, their biomass and activity were relatively low, resulting in a low TNRE. In addition, the nitrate produced by the metabolic activity of the still-developing bacterial community may represent a comparatively large fraction of the total nitrogen species. As the biofilm gradually matured (toward the 35th day), anammox bacteria became the dominant bacterial community, their metabolic activities increased, which formed a symbiotic relationship with denitrifying bacteria, etc., leading to an improvement and stabilization of the system’s removal nitrogen performance.

Based on an influent NH_4_^+^-N concentration of 35 mg/L, the optimal theoretical NO_3_^−^-N production for anammox was determined to be 9.80 mg/L. Among the reactors, the NO_3_^−^-N concentration in R1 was the closest to the theoretical value, indicating that the anammox performance in R1 was optimal. This is further supported by the trend in the ratio of anaerobic ammonium oxidation to nitrogen removal (RA), as illustrated in Figure 3D. However, the effluent NO_3_^−^-N concentrations in all three reactors were lower than the theoretical value, suggesting that denitrifying bacteria in each reactor reduced part of the NO_3_^−^-N to N_2_. Variations in effluent concentrations of NH_4_^+^-N, NO_2_^−^-N, and TNRE demonstrated that the anammox biofilms in R1 and R2 exhibited better immobilization and growth than those attached to the polyethylene ring carrier in R3. The main distinction lay in stability, although both ultimately achieved a TNRE exceeding 90%, indicating that the polyethylene ring carrier was less efficient in enriching anammox microorganisms than the former two carriers. The fluctuation in effluent NO_2_^−^-N concentrations across the reactors between days 90 and 105 was likely attributed to the presence of trace oxygen within biofilm interstices, which sustained nitrification activity. AOB was responsible for converting NH_4_^+^-N to NO_2_^−^-N.

These nitrogen removal efficiency in typical cycle indicate that the anammox performance was highest in R1, followed by R2, and lowest in R3, whereas nitrification was most pronounced in R3. The slight decrease of TNRE in reactor R1 during the final stage indicated the presence of nitrifying bacteria. This aligns with the fact that the highly porous internal structure of the loofah sponge carrier provides ample space for biofilm formation and retains trace amounts of oxygen within the matrix, thereby supporting limited nitrification. Although the RA began to decline in the middle and later stages, it consistently maintained the highest ratio among the three reactors. This indicates that the activity of AnAOB was highest in R1, resulting in optimal nitrogen removal performance. The initial decline in RA is likely attributable to competition with other beneficial functional microorganisms. This competition, along with the subsequent dynamic balance in microbial distribution, further supports the conclusion that nitrogen removal was most effective in R1. The TNRR values for the three reactors in the typical cycle experiments also indicated a stronger treatment capacity for R1. In summary, R1 (loofah sponge carrier) exhibited outstanding performance during typical cyclic experiments in terms of nitrogen removal efficiency, RA, TNRE, TNRR, operational stability, and by-product control. In contrast, R2 (polyurethane sponge carrier) had issues of delayed NO_2_^−^-N removal and inadequate stability in the later phase; R3 (polyethylene carrier) showed subpar performance across all indicators throughout the process, with incomplete substrate removal, and was unfavorable for microbial attachment and metabolism. In comparison with R2 and R3, the loofah sponge in R1 held significant advantages in AnAOB enrichment and nitrogen removal.

The morphological differences in biofilms among the three carriers are primarily attributed to their inherent structural properties by SEM: the natural fibrous interwoven structure of the loofah sponge carrier forms a three-dimensional network with a dense surface and sparse interior, creating a densely porous spatial structure that provides abundant initial attachment sites for microorganisms. Biofilms tend to aggregate at fiber intersections and form stable structures; the polyurethane sponge carrier is an artificially synthesized material with regularly arranged pores. Microorganisms and EPS tightly encase the sponge fibers, forming a thick, homogeneous biofilm layer with clear boundaries. The biofilm is mainly attached to the sponge framework, presenting as irregular sheets or fragments, while the carrier framework has a regular foam structure; the surface of the polyethylene carrier is smooth or slightly rough, making it difficult for microorganisms to form a uniform and continuous covering layer.^24^ Microorganisms are mostly attached as single cells or small clusters, although localized regions exhibit greater attachment thickness, resulting in uneven microbial distribution and potential poor stability. In summary, analysis of SEM images at different magnifications demonstrates that the loofah sponge carrier exhibits significant advantages in biofilm formation among the three carriers: its natural fibrous network structure provides sufficient attachment sites, ensuring uniform microbial coverage and tight adhesion to the carrier, thus achieving high integrity and stability of biofilm formation; the porosity formed by the internal fibers offers ample growth space for microorganisms, facilitating the establishment of a stable “microbe-EPS” composite structure; the formation of a microaerobic environment within the carrier promotes the co-dominant colonization of various functional bacteria, leading to beneficial microenvironmental differentiation. In contrast, the polyurethane sponge carrier performs moderately, with uniform biofilm formation and high microbial biomass, but poses potential risks such as excessive compactness, susceptibility to pore clogging, and incomplete utilization of internal pores. The polyethylene ring carrier exhibits the poorest performance, characterized by uneven and discontinuous biofilm coverage and poor stability.

These trends of EPS align with previous similar studies. During the day 0–80, microorganisms secreted large amounts of EPS, particularly TB-EPS, which acts as a “structural framework” for biofilm adhesion and architecture. In the maturation-stable stage (day 80–110), the biofilm structure was largely established, and EPS secretion shifted from “construction” to “maintenance,” approaching a dynamic equilibrium. Across all stages and layers, the increase in PN—both in magnitude and absolute content—was far greater than that of PS. This is because hydrophobic proteins play a crucial role in enhancing the structural stability of biofilms. The rise in the PN/PS ratio serves as a key indicator of biofilm maturation. Among the three EPS layers, S-EPS changed the least, as it primarily functions in dispersal, lubrication, and initial signaling. LB-EPS, serving as a “buffer layer” between TB-EPS and the external environment, exhibited moderate change. The TB-EPS layer, as the main structural framework supporting the overall biofilm morphology, underwent the most pronounced changes in EPS concentration and was the primary site of PN accumulation, directly determining the biofilm’s mechanical strength and stability.

In R1, biofilms showed the greatest increase in EPS concentration. This is attributed to the loofah sponge carrier, composed of natural fibers with a dense network structure, high specific surface area, and good hydrophilicity, which provided an optimal habitat for microorganisms and strongly stimulated EPS secretion—especially PN—to build a stable microenvironment. The surface contains lignin and hemicellulose, offering additional hydrogen-bonding sites and promoting PN enrichment. The release of degradable components resulted in low-molecular weight organics, perceived by bacteria as a “carbon surplus,” triggering energy-storing EPS secretion. In R2, the change in biofilm EPS concentration was second only to R1. Its carrier, polyurethane sponge, is a synthetic porous material with uniform pores and a very high specific surface area, effectively promoting adhesion and EPS secretion. However, its biocompatibility is slightly inferior to natural loofah, and overly dense pores may cause internal mass transfer resistance, affecting microbial activity and potentially leading to “active hollowing.” In R3, biofilm EPS concentration changed the least. The polyethylene ring carrier has a smooth, hydrophobic surface, which is less conducive to initial adhesion. Microorganisms required more time and more EPS to “modify” the surface, resulting in the lowest EPS accumulation rate and increment.

These disparities are attributed to the distinct microbial attachment effects of the different carriers employed in each reactor. The results from one-way analysis of variance (ANOVA) combined with Tukey’s test indirectly demonstrate that the loofah sponge carrier in reactor R1 exhibits a significant advantage in terms of microbial enrichment and biofilm formation.

Based on 16S rRNA gene sequencing data, the abundance of functional microorganisms in reactor R1 exhibited a slight increase, yet this change was inconsistent with its outstanding nitrogen removal performance as reported in the preceding sections. Similarly, the relative abundance of AnAOB in reactor R3 increased from 34.59% to 56.40%, whereas the corresponding nitrogen removal efficiency displayed a trend completely contrary to the variation in microbial abundance. Therefore, changes in the abundances of *Planctomycetota* and *Candidatus Brocadia* alone cannot reflect variations in their nitrogen removal capacity, which is further confirmed by subsequent metagenomic analysis. According to previous studies, a high abundance of a specific bacterium only indicates a greater quantity of that bacterium; its physiological state and activity cannot be fully represented by the abundance data obtained from 16S rRNA sequencing, although the two typically show a consistent trend in most cases.^2^^,^^8^ Additionally, this discrepancy may be influenced by factors such as mass transfer within the carrier, inhibitory substances, or microbial interactions.^40^ Moreover, the variation trends in the abundances of other phyla may provide another potential explanation for this anomaly. Additionally, this discrepancy may be influenced by factors such as mass transfer within the carrier, inhibitory substances, or microbial interactions.^40^ Moreover, the variation trends in the abundances of other phyla may provide another potential explanation for this anomaly.

Both Proteobacteria and Bacteroidota reached relatively high levels in R1: the increased abundance of Proteobacteria showed a consistent trend in at least R2 and R3, a normal phenomenon since Proteobacteria act as denitrifying bacteria that coexist with AnAOB. This observation has been reported in numerous studies.^41^^,^^42^ In contrast, the abundance of Bacteroidota continued to increase in R1, which was completely opposite to the pattern in the other two reactors. This change may be associated with organic matter degradation and the provision of metabolic products to support AnAOB processes, forming a synergistic metabolic network among multiple microbial communities to facilitate nitrogen transformation.^43^ This is presumably the valid explanation for the observed phenomenon.

Despite the high abundance of AnAOB in R3, its nitrogen removal efficiency was the poorest. This situation can be attributed to factors including inadequate mass transfer within the carrier itself, and uneven, excessively thick biofilms that result in reduced activity of subsurface-layer microorganisms. Furthermore, competitive or inhibitory bacterial communities may exist in R3. As shown in Figure 7B, the abnormal increase in the abundance of certain bacterial genera (e.g., *WWE3* and *SC-I-84*) in R3 suggests that these bacteria may have inhibited the activity of functional AnAOB. Overproliferation of AnAOB may also have occurred, leading to an imbalance in the microbial community structure and a lack of other functional bacteria, thereby precluding the formation of a synergistic effect.^44^

In conclusion, the relatively lower abundance of functional microbial communities did not negatively impact the nitrogen removal efficiency in reactor R1, which utilized loofah sponge as the carrier. On the contrary, it formed a stark contrast to the high abundance coupled with high efficiency in R2 and the high abundance with low efficiency in R3. This discrepancy demonstrated that the relative quantity of microorganisms on the carrier is not the sole determinant of their functional activity. A comprehensive analysis of the final nitrogen removal effect must consider the combined influence of the carrier’s distinct structure, the intrinsic activity of the functional microorganisms, and the synergistic interactions within the entire microbial community. The lower microbial abundance in R1, paired with its superior nitrogen removal efficiency, provides compelling evidence that the functional microorganisms on the loofah sponge carrier exhibit a higher specific activity. The unique structure of the loofah sponge fostered a more favorable local microbial habitat, promoting the survival and metabolic activity of the target functional species. Consequently, its unit nitrogen removal capacity per microorganism is superior to that of the two synthetic carriers.

These enzyme abundance data in anammox pathway indicate that reactor R1 had an absolute advantage in the conversion of nitrite to nitric oxide, followed by R2; in the conversion of nitric oxide to hydrazine, R2 performed the best, with R1 ranking second; and in the hydrazine dehydrogenation step, R2 was superior, while R1 was the lowest—though the *hdh* abundance in R1 was only 2 × 10^−7^ lower than that in R3. The gene abundance patterns observed here are highly consistent with the concentration trends of ammonia nitrogen in the effluent during the typical cycle (where R2 performed optimally) and the nitrite concentration trends (where R1 was superior).

Variations in these denitrification pathway genes suggest that denitrifying bacteria hold a distinct competitive advantage within the microbial community of reactor R1. It is well established that AnAOB cannot yet be cultured as pure cultures, and the associated denitrifying bacteria also play a crucial role in the overall nitrogen removal process. Therefore, the highest abundance of denitrification-related enzymes in R1 may also contribute to its superior nitrogen removal performance. Furthermore, since some enzymes in the nitrogen cycle are shared across different pathways, *napA/B* abundance does not indicate the dominance of the dissimilatory nitrate reduction pathway.^45^ Data from the other two pathways indicate that the assimilatory nitrate reduction pathway in reactor R1 was the weakest among the three reactors, and that the nitrogen fixation pathway was not detected in the nitrogen cycle of R1.

In summary, among the six major nitrogen cycle metabolic pathways, functional enzyme abundance data demonstrate that two key nitrogen removal pathways—the anammox and denitrification pathways—were dominant in reactor R1’s microbial community, while the other four pathways were expressed at relatively low levels. This observation confirms the rationality of the negative correlation between R1’s microbial community abundance sequencing results and the reactor’s effluent residual nitrogen concentration. Furthermore, it reveals at the genetic level how the natural loofah sponge carrier used in reactor R1 achieved superior nitrogen removal performance by high abundance expression of key functional genes, despite a relatively low enrichment of AnAOB.

Transport proteins play a central role in microbial physiology by transporting essential nutrients and energy sources into cell from external environment.^46^^,^^47^ The *amt* family transporter protein is responsible for transporting extracellular NH_4_^+^ across the membrane into the cell. It is a key protein for AnAOB to obtain the core substrate, ammonia. Regarding this key ammonium transporter, the biofilm on the loofah sponge carrier maintained the greatest advantage. However, *amt* abundance was also relatively high in the biofilms of the other two carriers. The abundance level of the *amt* gene corresponded to the NH_4_^+^-N substrate demand of AnAOB, such as *Candidatus Brocadia*.

Iron serves as a cofactor for various enzymes involved in the anammox process. Therefore, iron-related transporter proteins were also a key focus of this study. The *FeoB* family and the iron/lead transporter (*ILT*) family are the major iron transporter proteins identified in the metagenomic data.^48^^,^^49^ The *FeoB* family functions as a specific transporter for Fe^2+^ and constitutes the primary pathway for iron uptake under anaerobic conditions. In contrast, *ILT* family proteins mainly mediate the transmembrane transport of Fe^3+^/Fe^2+^, facilitating the transport of iron ions from the extracellular to the intracellular environment and thereby providing the iron necessary for anammox reactions. The ATP-binding cassette (*ABC*) superfamily is one of the largest and most evolutionarily conserved families of membrane transport proteins, present across all domains of life from bacteria to humans. These proteins utilize energy derived from ATP binding and hydrolysis to actively transport a diverse range of substrates across biological membranes. In AnAOB systems, members of the ABC superfamily have been shown to be involved in the uptake of iron carrier complexes, indirectly supplementing the supply of iron cofactors.^50^^,^^51^

In conclusion, the gene expressions of the two key proteins (*amt* and *FNT*) in R1 and R2 indicated that the transport capacities of substrates required for the anammox process were comparable in their own reactor. Consistent with the low expression levels of *amt* and *FNT* transport proteins, the R3 reactor exhibited unsatisfactory nitrogen removal performance compared to the other two reactors. In reactor R1, four of the five auxiliary transport proteins analyzed (*FeoB*, *ILT*, *F-ATPase*, and *FixABCX*) showed a clear genetic advantage at the transport protein level. This finding supports the conclusion that, among the three carriers tested, the loofah sponge carrier contributes most significantly to the anammox reaction. These genes expression of primary transport proteins in loofah sponge carrier biofilm were consistent with the previously observed trends in functional enzyme abundances and effluent nitrogen concentrations, combined with the bacterial abundance data from 16S rRNA sequencing. These results further explain why a high nitrogen removal efficiency was achieved despite the relatively low enrichment abundance of AnAOB in the biofilm on the loofah sponge carrier, which confirms that the relationship between bacterial enrichment quantity and activity does not exhibit a completely positive correlation.

Results from the co-expression analysis of all two pairs of functional genes are as follows: negative correlations suggest potential functional trade-offs between the hydrazine metabolism and nitrite reduction pathways, whereas positive correlations reflect the tight co-regulation of hydrazine dehydrogenase/hydrazine synthase and nitrite reductase paralogs, respectively. The data points from reactor R1 (red dots) consistently clustered at higher gene abundance levels for hdh, hzs, and nirK, while those from R3 (green triangles) exhibited lower abundance, and R2 (blue squares) occupied an intermediate position. This observation suggests that reactor R1 maintains a more transcriptionally active state for key anammox genes. The total abundance of core functional genes varied significantly across reactors. This trend directly aligns with the superior nitrogen removal performance observed in R1, as higher functional gene expression likely translates to enhanced metabolic capacity. The co-expression correlation heatmap indicate a high degree of similarity in their overall gene expression patterns. Crucially, when combined with the scatterplot data showing R1’s higher absolute gene abundance, R1’s distinct profile-featuring elevated expression of the rate-limiting *hdh* and *hzs* genes-positions it as the most metabolically active reactor. The strong correlation between R2 and R3 suggests they share a more similar, yet less active, functional gene network compared to R1. Collectively, the co-expression analysis demonstrates that reactor R1, equipped with the loofah sponge carrier, fosters a more active and functionally distinct expression of key anammox functional genes. The higher absolute abundance of core genes in R1, combined with its unique correlation profile, provides a molecular-level explanation for its superior microbial enrichment, biofilm formation, and nitrogen removal performance compared to R2 and R3.

The correlation analysis highlights that the superior nitrogen removal performance of R1 is closely tied to the elevated abundance of core functional genes. The strong positive correlation between gene abundance and TNRE (r = 0.68) provides compelling evidence that the loofah sponge carrier in R1 fosters a more metabolically active microbial community, characterized by enhanced expression of genes critical to anammox. This, in turn, translates to lower effluent nitrogen concentrations and higher overall treatment efficiency, positioning R1 as the most effective reactor configuration.

Summarizing the above research findings, a radar chart was employed to visually depict the nitrogen removal performance of the three reactors and the microbial community characteristics of the biofilms.

**Figure 12:**
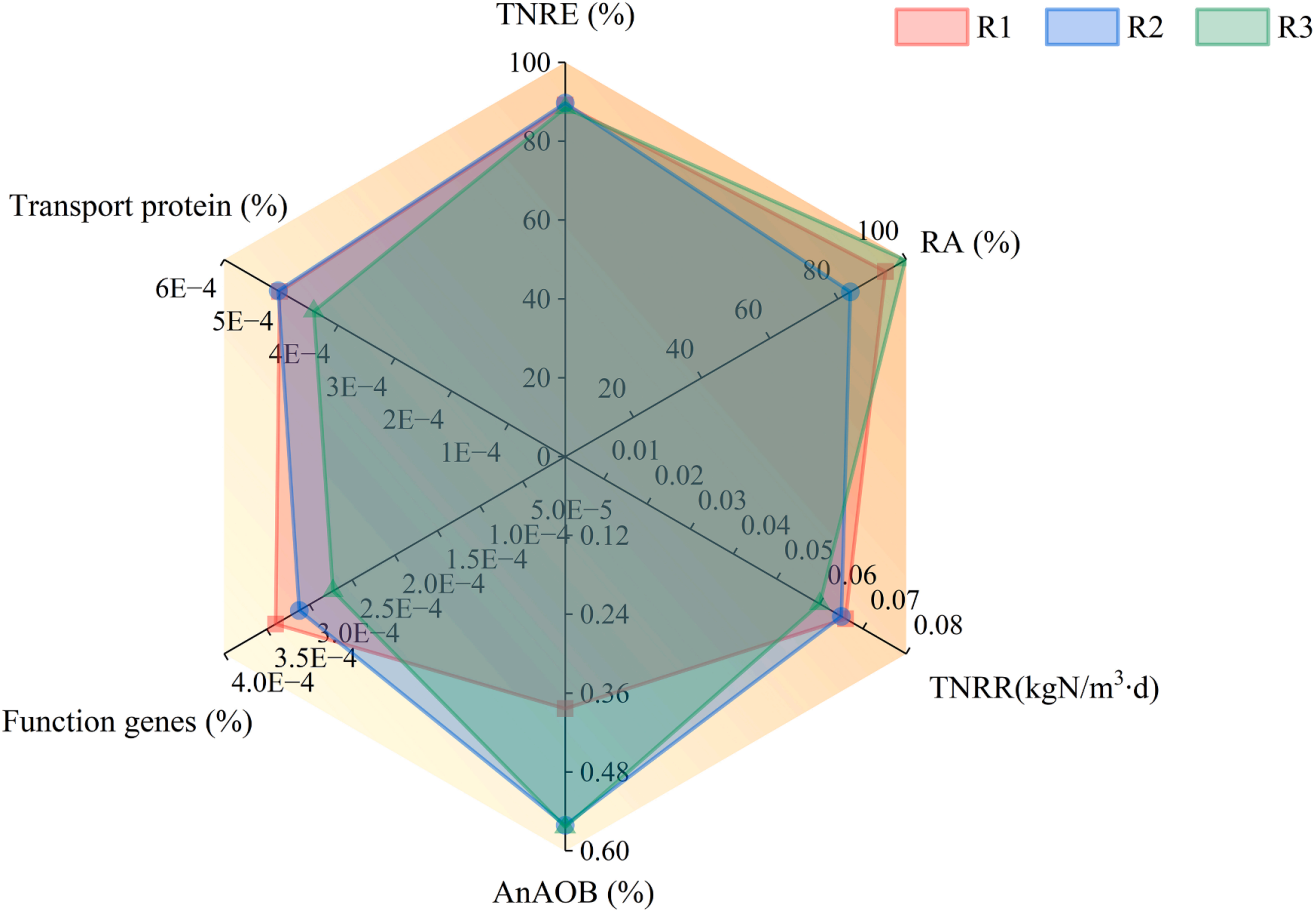
Comprehensive radar chart of reactor performance and microorganism functional traits across three anammox reactors

As shown in Figure 12, the radar chart provides a comprehensive comparison of key performance indicators and microorganism functional traits across reactors R1, R2, and R3. R1 achieved the highest TNRE, outperforming R2 and R3, this confirms R1’s superior capacity for nitrogen removal. R1 exhibited the highest TNRR, indicating a more rapid nitrogen transformation rate compared to R2 and R3. R1 displayed the highest relative abundance of functional genes, reflecting a more transcriptionally active microbial community. R1 had a higher transport protein abundance than R2 and R3, suggesting enhanced nutrient uptake and metabolic efficiency. The radar chart analysis confirms that reactor R1, with the loofah sponge carrier, outperforms R2 and R3 across a large proportion of key metrics. The higher TNRE and TNRR in R1 are directly linked to its elevated functional gene expression and transport protein levels, which collectively enhance metabolic activity and nitrogen removal efficiency. These results demonstrate that the carrier in R1 fosters a more robust and functionally active microbial community, making it the most effective configuration for anammox-based wastewater treatment.

To our knowledge, this study was the first to explore the use of loofah sponge as an immobilized carrier for AnAOB biofilm and nitrogen removal in low-nitrogen wastewater. In the R1 reactor, the loofah sponge carrier biofilm achieved a TNRE exceeding 90% and a peak TNRR of 0.067 kgN/(m^3^·d). Long-term and typical cycle experiments across three reactors demonstrated that the anammox biofilm on the loofah sponge carrier achieved the optimal nitrogen removal performance. This analysis was based on: (1) the uniformity and thickness of the microbial biofilm morphology; (2) the abundance of key bacterial groups and genera involved in anaerobic ammonium oxidation; and (3) the gene expression of principal anammox functional enzymes along with two key and five auxiliary transport proteins. With the exception of relative microbial community abundance, it demonstrated absolute superiority over the other two carriers in all other aspects. This study demonstrates that loofah sponge is a high-efficiency, green and sustainable biofilm carrier suitable for anammox processes. Future research can focus on the surface modification optimization of loofah sponge to further improve its microbial adhesion performance and service life, and carry out pilot-scale tests to lay a foundation for its practical engineering application.

### Limitations of the study

This research was carried out under laboratory-scale conditions, and the long-term operating stability of the loofah sponge carrier under complex actual wastewater components and variable environmental conditions, as well as the economic benefit evaluation of large-scale engineering application, still need to be further verified.

## Resource availability

### Lead contact

Requests for further information and resources should be directed to and will be fulfilled by the lead contact, Yongzhi Chen (476411589@qq.com; cyz@lzjtu.edu.cn). The lead contact is responsible for communicating with the journal before and after publication, responding to requests regarding materials and resource sharing, and ensuring the accessibility of all materials, datasets, and protocols used in the manuscript.

### Materials availability

In this study, three kinds of biofilm carrier samples were obtained from network shop, with relevant specifications consistent with the chemical composition and structural characteristics reported in existing studies. Standard laboratory reagents used in the study are commercially available and do not require special access restrictions.

### Data and code availability

Data requests should be directed to and will be fulfilled by the lead contact. The study in this article does not report original code. Additional information will be made available by the lead contact upon request.

## Acknowledgments

This work was supported by 10.13039/501100018554Science and Technology Program of Gansu Province (24CXNA029), Department of Education of Gansu Province: Major cultivation project of scientific research innovation platform in university (2024CXPT-14). The Key Research and Development Program of Gansu Province—Industrial Project under grant (25YFGA054).

## Author contributions

J.L., writing – original draft and investigation; J.F., review and editing; H.L., writing – review and editing, investigation, and funding acquisition; X.L., formal analysis; Z.T., methodology and software; X.W., investigation and data curation; F.A., data curation; Y.C., writing – review and editing and funding acquisition.

## Declaration of interests

The authors declare that they have no known competing financial interests or personal relationships that could have appeared to influence the work reported in this paper.

## STAR★Methods

### Key resources table

**Table:** 

REAGENT OR RESOURCE	SOURCE	IDENTIFIER
**Bacterial and virus strains**		

Anammox bacteria (*Candidatus_Brocadia*)	Laboratory-enriched anammox sludge	NCBI:txid174632

**Chemicals, peptides, and recombinant proteins**		

Ammonium chloride (NH_4_Cl)	Aladdin	CAS: 12125-02-9
Sodium nitrite (NaNO_2_)	Aladdin	CAS: 7632-00-0
Potassium phosphate monobasic (KH_2_PO_4_)	Aladdin	CAS: 7778-77-0
Magnesium sulfate (MgSO_4_)	Aladdin	CAS: 7487-88-9
Calcium chloride (CaCl_2_)	Aladdin	CAS: 10043-52-4
Sodium bicarbonate (NaHCO_3_)	Aladdin	CAS: 144-55-8
Phenol (C_6_H_6_O)	Aladdin	CAS: 108-95-2
Sulfuric acid (H_2_SO_4_)	Aladdin	CAS: 7664-93-9

**Deposited data**		

16S rRNA sequencing	Magigene	RRID: SCR_005397
Metagenomic sequencing	Magigene	RRID: SCR_005397

**Experimental models: Organisms/strains**		

Anammox bacteria (*Candidatus_Brocadia*)	Laboratory-enriched anammox sludge	NCBI:txid174632

**Software and algorithms**		

Origin 2024	OriginLab Corporation	RRID: SCR_014212
R	R Foundation for Statistical Computing	RRID: SCR_001905

### Experimental model and study participant details

Not applicable. This study does not involve vertebrate animals, human participants, human tissues, or standard laboratory biological models.

### Method details

#### Reactor configuration and operation

Two laboratory-scale anaerobic reactors were used in this study: a 5 L continuous-flow column reactor for Anammox biofilm cultivation, and a 1 L anaerobic sequencing batch reactor (ASBR) for nitrogen removal performance evaluation.^52^

The 5 L continuous-flow column reactor was fabricated from acrylic, with a height of 500 mm, outer diameter of 150 mm, and wall thickness of 5 mm. Influent and effluent pipelines with an inner diameter of 8 mm were installed at 100 mm intervals along the vertical height. The reactor was equipped with a mechanical stirrer operated at 120 rpm and a fixed bracket for biofilm carriers. Influent was pumped upward from the bottom using a peristaltic pump, and effluent was discharged through an overflow outlet at the top. The 1 L ASBR was a cylindrical glass vessel with a height of 220 mm and diameter of 100 mm, sealed with a lid. Mixing was provided by a magnetic stirrer at 120 rpm. Influent and effluent were controlled via a peristaltic pump at the top, with a water exchange ratio ≥95%.

Biofilm carriers were securely fixed inside each reactor. All reactors were automatically controlled by a programmable logic controller (PLC). Reactor temperature was maintained at 28°C–35°C using a temperature controller, and pH was stabilized at 7.0–8.0 by dosing NaHCO_3_. The experiment was conducted in two consecutive phases with operating parameters listed in Table 2.

#### Influent preparation, inoculation sludge and biofilm carriers

Synthetic low-nitrogen wastewater was prepared by adding analytical-grade NH_4_Cl (0.1338 g/L) and NaNO_2_ (0.2277 g/L) to tap water, yielding influent concentrations of 35 mg/L NH_4_^+^-N and 46.2 mg/L NO_2_^−^-N throughout the experiment. Additional nutrients including KH_2_PO_4_, MgSO_4_, CaCl_2_, NaHCO_3_, and two trace element solutions were supplemented to simulate actual wastewater and buffer pH.^53^

Anammox seed sludge was cultivated in the laboratory from aerobic activated sludge collected from the aeration tank of a municipal wastewater treatment plant in Lanzhou. The inoculated sludge had a mixed liquor suspended solids (MLSS) concentration of 6433 mg/L and mixed liquor volatile suspended solids (MLVSS) of 4285 mg/L.^53^ The volume ratio of biofilm carrier to reactor liquid was approximately 1:5.^46^

#### Three types of carriers were tested

Loofah sponge: cut transversely into 2 cm-wide rings, fixed vertically on screws; 3 pieces per screw, 4 screws per 1 L reactor (total 12 carriers).

Polyurethane sponge: cut into 2 cm × 2 cm cubes, fixed identically; 24 carriers per 1 L reactor.

Polyethylene carriers: fixed identically; 40 carriers per 1 L reactor.

Before use, loofah sponges were rinsed thoroughly with tap water to remove surface impurities, dried to constant weight at 60°C, and autoclaved to eliminate indigenous microbes.

#### Sample collection and analytical methods

Effluent samples were collected at fixed intervals: every 48 h during biofilm cultivation, and every 24 h during the nitrogen removal phase. Concentrations of NH_4_^+^-N, NO_2_^−^-N, NO_3_^−^-N, MLSS, and MLVSS were measured according to standard methods (APHA, 1998). Total nitrogen (TN) was calculated as the sum of NH_4_^+^-N, NO_2_^−^-N, and NO_3_^−^-N. Dissolved oxygen (DO), pH, and temperature were monitored online using a WTW Multi 3620 m (Germany).

#### Scanning electron microscopy (SEM) observation

Biofilm morphology on carriers was observed using scanning electron microscopy. Triplicate carrier samples were collected on day 110, cut into small pieces, vacuum-dried, mounted on conductive adhesive tabs, and sputter-coated with gold. Microbial morphology and biofilm structure were examined using a scanning electron microscope.

#### Extraction and analysis of extracellular polymeric substances (EPS)

EPS was extracted using a modified thermal extraction method, separating into three fractions: soluble EPS (S-EPS), loosely bound EPS (LB-EPS), and tightly bound EPS (TB-EPS).

Polysaccharide (PS) content was determined by the phenol–sulfuric acid method. Protein (PN) content was measured by the Coomassie brilliant blue method. Slurry samples (15 mL) were collected in triplicate at days 0, 80, and 110 for EPS extraction and quantification.

#### Microbial high-throughput sequencing (16S rRNA gene)

Biofilm samples were collected on day 1 (initial) and day 105 (stable operation) from 1 L reactors. Biofilm was stripped from carriers, and 50 mL of the sludge mixture was centrifuged at 4000 rpm for 5 min. Pellets were stored at −80°C until absolute quantitative 16S rRNA high-throughput sequencing.

Genomic DNA was extracted using a high-throughput sequencing kit. DNA purity and concentration were verified using Nanodrop One and Qubit assays, respectively. The V3–V4 hypervariable region of the 16S rRNA gene was amplified using universal primers 338F (ACTCCTACGGGAGGCAGAG) and 806R (GGACTACHVGGGTWTCTAAT). Amplicon libraries were sequenced on an Illumina PE250 platform by Guangdong Magigene Biotechnology Co., Ltd. (Guangzhou, China).

#### Metagenomic sequencing and analysis

Metagenomic sequencing was performed to analyze key functional genes related to Anammox metabolism. Biofilm samples were collected on days 1 and 105, pretreated, and stored at −80°C as described above.

Qualified DNA was fragmented randomly using an ultrasonic disruptor, and libraries were constructed and quality-checked. Sequencing was performed on an Illumina NovaSeq 6000 platform with PE150 mode. Raw reads were converted to FASTQ format and quality-filtered using Fastp. Clean reads were used for *de novo* metagenomic assembly, gene prediction, clustering, and redundancy removal.

For taxonomic annotation, Unigenes were aligned against the NCBI-NR database; Metaphlan was used for rapid taxonomic classification. Functional annotation was performed by alignment against KEGG, eggNOG, and CAZy databases.^46^

#### Calculation formulas

##### Total nitrogen removal efficiency (TNRE)

The TNRE is removal efficiency of all inorganic nitrogen in reactor, which was calculated as Equation 1.

(Equation 1) $$TNRE\frac{TN_{\inf}-TN_{eff}}{TN_{\inf}}\text{,}$$

where TN_inf_ and TN_eff_ were the total inorganic nitrogen concentration of influent and effluent in reactor.

##### Ratio of anammox nitrogen removal contribution (RA)

RA is an important index, it is the contribution ratio of nitrogen removal by Anammox, which was calculated as Equation 2.

(Equation 2) $$RA=\frac{2.06\times \left({NH}_{4}^{+}-N_{\inf}-{NH}_{4}^{+}-N_{eff}\right)}{\left({NH}_{4}^{+}-N_{\inf}-{NH}_{4}^{+}-N_{eff}\right)+\left({NO}_{2}^{-}-N_{\inf}-{NO}_{2}^{-}-N_{eff}\right)+{NO}_{3}^{-}-N_{eff}}\text{.}$$

##### Total nitrogen loading rate (TNLR)

The TNLR is volume load ratio of total inorganic nitrogen unit time in reactor, which was calculated as Equation 3.

(Equation 3) $$TNLR=\frac{{TN}_{\inf}\times 24}{1000\times T}\text{,}$$

where T was the entire time of one cycle.

##### Total nitrogen removal rate (TNRR)

The TNRR is removal load ratio of total inorganic unit time nitrogen in reactor, which was calculated as Equation 4.

(Equation 4) $$TNRR=\frac{\left({TN}_{\inf}-{TN}_{eff}\right)\times 24}{1000\times T}\text{.}$$

### Quantification and statistical analysis

One-way analysis of variance (ANOVA) was used to evaluate significant differences in total EPS concentrations among three reactors (R1, R2, R3) across different EPS fractions (S-EPS, LB-EPS, TB-EPS) and time points (0, 80, 110 days). When significant effects were detected (*p* < 0.05), Tukey’s honestly significant difference (HSD) post-hoc test was used for multiple pairwise comparisons. All statistical analyses were performed using Origin 2024. Data are presented as mean ± standard error of the mean (SEM) with *n* = 3. Different lowercase letters above bars indicate significant differences (*p* < 0.05) between reactors at the same fraction and time point.
